# Pharmacogenetics-Based versus Conventional Dosing of Warfarin: A Meta-Analysis of Randomized Controlled Trials

**DOI:** 10.1371/journal.pone.0144511

**Published:** 2015-12-16

**Authors:** Changcheng Shi, Wei Yan, Gang Wang, Fei Wang, Qingyu Li, Nengming Lin

**Affiliations:** 1 Department of Clinical Pharmacy, Hangzhou First People’s Hospital, Hangzhou, Zhejiang Province, China; 2 Department of Clinical Pharmacology, Hangzhou Translational Medicine Research Center, Hangzhou, Zhejiang Province, China; 3 Affiliated Hangzhou Hospital, Nanjing Medical University, Hangzhou, Zhejiang Province, China; 4 The first Affiliated Hangzhou Hospital, Zhejiang Chinese Medical University, Hangzhou, Zhejiang Province, China; University of Naples Federico II, ITALY

## Abstract

**Background:**

Recently, using the patient’s genotype to guide warfarin dosing has gained interest; however, whether pharmacogenetics-based dosing (PD) improves clinical outcomes compared to conventional dosing (CD) remains unclear. Thus, we performed a meta-analysis to evaluate these two strategies.

**Methods:**

The PubMed, Embase, Cochrane Library, China National Knowledge Infrastructure (CNKI), Chinese VIP and Chinese Wan-fang databases were searched. The Cochrane Collaboration’s tool was used to assess the risk of bias in randomized controlled trials (RCTs). The primary outcome was time within the therapeutic range (TTR); the secondary end points were the time to maintenance dose and time to first therapeutic international normalized ratio (INR), an INR greater than 4, adverse events, major bleeding, thromboembolism and death from any cause.

**Results:**

A total of 11 trials involving 2,678 patients were included in our meta-analysis. The results showed that PD did not improve the TTR compared to CD, although PD significantly shortened the time to maintenance dose (MD = -8.80; 95% CI: -11.99 to -5.60; P<0.00001) and the time to first therapeutic INR (MD = -2.80; 95% CI: -3.45 to -2.15; P<0.00001). Additionally, PD significantly reduced the risk of adverse events (RR = 0.86; 95% CI: 0.75 to 0.99; P = 0.03) and major bleeding (RR = 0.36; 95% CI: 0.15 to 0.89, P = 0.03), although it did not reduce the percentage of INR greater than 4, the risk of thromboembolic events and death from any cause. Subgroup analysis showed that PD resulted in a better improvement in the endpoints of TTR and over-anticoagulation at a fixed initial dosage rather than a non-fixed initial dosage.

**Conclusions:**

The use of genotype testing in the management of warfarin anticoagulation was associated with significant improvements in INR-related and clinical outcomes. Thus, genotype-based regimens can be considered a reliable and accurate method to determine warfarin dosing and may be preferred over fixed-dose regimens.

**Trial Registration PROSPERO:**

Database registration: CRD42015024127.

## Introduction

Warfarin, a commonly used oral anticoagulant, has been proven to be effective in the treatment and prevention of thromboembolic events associated with atrial fibrillation (AF), deep vein thrombosis (DVT), pulmonary embolism (PE) and prosthetic heart valves [1]; however, warfarin’s narrow therapeutic window and inter- and intra-individual variability in dose requirements make warfarin dosing notoriously challenging in clinical practice [2,3]. Moreover, adverse events are common during the initial period of treatment before the maintenance dose is reached. Therefore, frequent monitoring of the patient’s international normalized ratio (INR) through periodic blood testing is warranted. Insufficient anticoagulation (INR lower than 2) increases the risk of thrombotic events, whereas overdosing (INR exceeding 3, particular above 4) confers a predisposition to bleeding [4,5].

Numerous physiological factors including age, body mass index, sex, race, dietary vitamin K intake and drug interactions are closely associated with warfarin dose requirement variations [6,7]. Although tremendous efforts have been made to improve warfarin dosing methods, no standardized regimen exists. During the last 10 years, research has shifted from a conventional dosing strategy to understanding the genetic factors of warfarin dosing. Indeed, several genes might be related to the activity and metabolism of warfarin, with the genotypes for cytochrome P450 2C9, CYP2C9 (related to the metabolism of S-warfarin) and the vitamin K epoxide reductase complex subunit 1 gene, VKORC1 (the molecular target of warfarin involved in the vitamin K cycle) gaining the most attention [8–11]. Recent data have suggested that warfarin dosing algorithms that combine genotypic information and clinical factors explain approximately half of the variation in the warfarin dose requirements [12–14]. These observations have raised interest in using genotype testing to guide the prescription of warfarin.

In recent years, a large number of genotype-based dosing models that incorporate genetic factors together with clinical characteristics have been developed; however, randomized controlled trials (RCTs) comparing pharmacogenetics-based dosing (PD) versus conventional dosing (CD) of warfarin have shown inconsistent outcomes. Thus, whether genotype-guided warfarin dosing can eventually improve clinical outcomes remains unclear. To address this issue, we performed a meta-analysis on all published RCTs to assess the effect of pharmacogenetics-based warfarin dosing in patients initiating warfarin therapy.

## Methods

### Search strategy

This systematic review was performed according to the Preferred Reporting Items for Systematic Reviews and Meta-Analyses (PRISMA) statement (S1 PRISMA Checklist) [15]. We systematically searched for unrestricted language articles included in the PubMed, Embase, Cochrane Library, China National Knowledge Infrastructure (CNKI), Chinese VIP, and Chinese Wan-fang databases from inception to March 2015. The literature search in PubMed was carried out following the strategy shown in Table 1. The details of the strategy used in other databases were shown in S1 Appendix. Reference lists of the relevant studies were searched for additional literature.

**Table 1:** Search strategy used in PubMed database.

Search number	Search query
#1	randomized controlled trial [pt]
#2	controlled clinical trial [pt]
#3	randomized [tiab]
#4	placebo [tiab]
#5	drug therapy [sh]
#6	randomly [tiab]
#7	trail [tiab]
#8	groups [tiab]
#9	#1 OR #2 OR #3 OR #4 OR #5 OR #6 OR #7 OR #8
#10	animal [mh] NOT humans [mh]
#11	#9 NOT #10
#12	genotype [mh]
#13	genes [mh]
#14	alleles [mh]
#15	polymorphism [mh]
#16	genetic [mh]
#17	pharmacogenetics [mh]
#18	genomics [mh]
#19	single nucleotide
#20	pharmacogenomics
#21	#12 OR #13 OR #14 OR #15 OR #16 OR #17 OR #18 OR #19 OR #20
#22	warfarin [mh]
#23	anticoagulants [mh]
#24	coumarin
#25	#22 OR #23 OR #24
#26	#11 AND #21 AND #25

### Inclusion/exclusion criteria

To be eligible, original studies were required to meet all of the following criteria: (1) RCTs design; (2) included patients at least 18 years old with an indication for anticoagulation; (3) comparison of pharmacogenetics-based versus conventional dosing of warfarin; and (4) sufficient outcomes to allow for calculation of effect sizes. Exclusion criteria included the following: (1) non-RCT studies; (2) studies that included participants with a history of treatment with warfarin and known maintenance dose; and (3) studies without data from a comparison group.

### Data extraction

Two investigators independently reviewed the full manuscripts of eligible articles, with each investigator blinded to the results of the other. The information extracted from each study included the first author, year of publication, study design, number of participants, patient characteristics (age, gender, race, indication), follow-up days, genotype (s) tested, warfarin dosing method, and outcomes. Any discrepancy was resolved through discussion.

### Risk of bias assessment

Quality assessment of the selected RCTs was performed with the Cochrane Collaboration’s tool for assessing the risk of bias [16]. This tool classifies studies as having a low, unclear, or high risk of bias across the following aspects: random sequence generation, allocation concealment, blinding of participants and personnel, blinding of outcome assessment, incomplete outcome data, selective reporting, and other sources of bias.

### Study outcomes

The primary outcome was the time within the therapeutic range (TTR). The secondary outcomes were an INR greater than 4, time to maintenance dose and the first target INR, adverse events during anticoagulation treatment, the frequency of major bleeding, thromboembolic events, and death from any cause.

### Statistical analysis

For dichotomous variables, the data were analyzed for a relative risk (RR) with 95% confidence intervals (CI) using the Mantel-Haenszel method. For continuous variables, the data were analyzed for the mean difference (MD) with a 95% CI using the inverse variance (IV) test. Fixed-effect or random-effect models were used for the meta-analysis, depending on the result of heterogeneity. Heterogeneity was explored using the I^2^ statistic. Significant heterogeneity existed for I^2^ > 50% [17]. When heterogeneity was confirmed, a sensitivity analysis was undertaken by successively excluding the studies. Subgroup analysis was performed according to the type of conventional regimen (fixed-dose regimen or non-fixed initial doses regimen). Publication bias was evaluated visually by inspecting funnel plots and statistically using Egger’s test [18]. All of the reported P-values were two-sided, with statistical significance set at 0.05. The statistical analyses were conducted using RevMan 5.3 (The Nordic Cochrane Centre, The Cochrane Collaboration, Copenhagen, Denmark).

## Results

### Study characteristics

**Fig 1:**
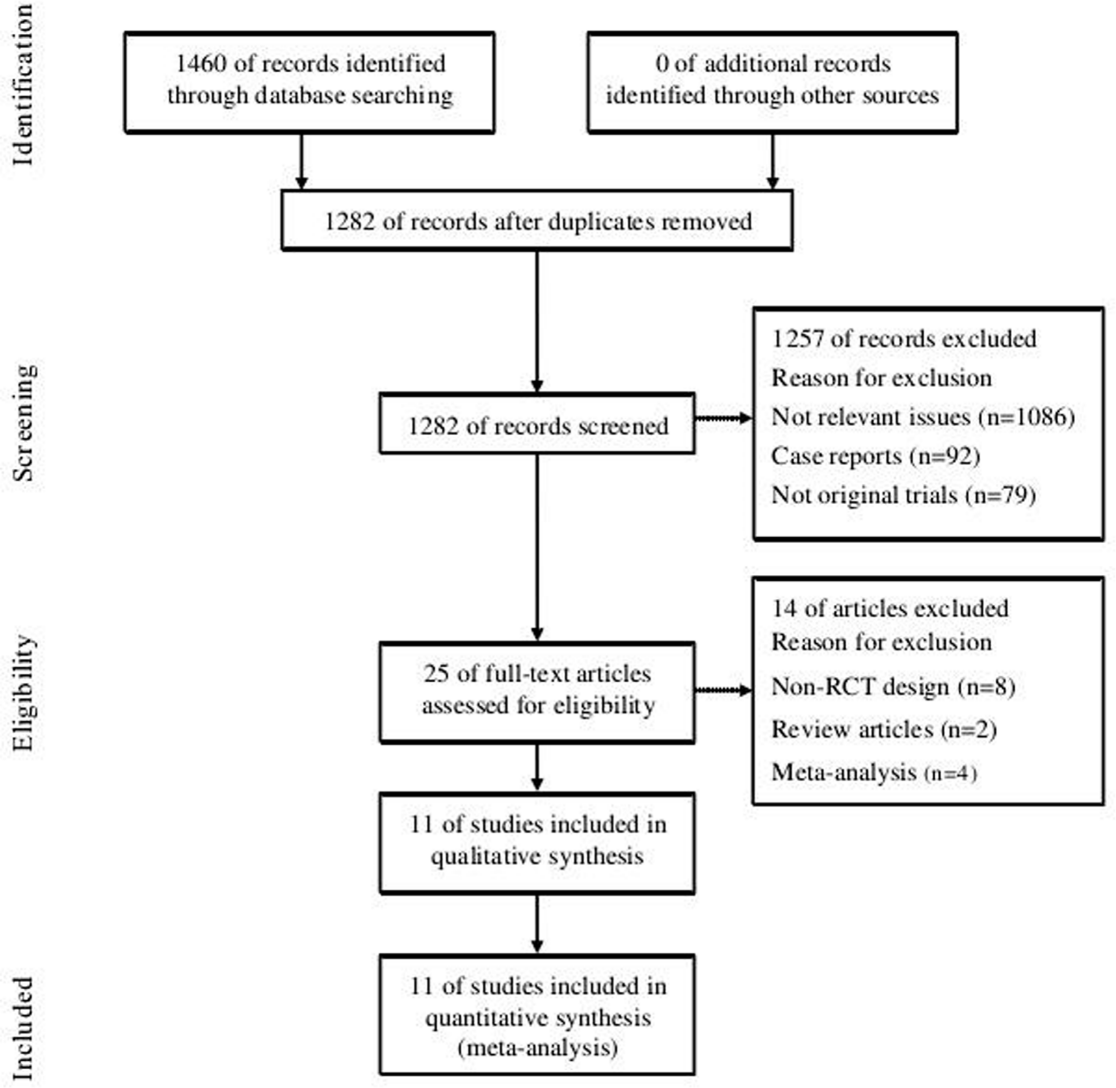
Flow diagram of the literature search and selection process.

Of the 1,460 articles found after the initial search, 11 studies involving 2,678 patients met the inclusion criteria [19–29]. The inclusion and exclusion of the RCTs for this meta-analysis are presented in a flow chart (Fig 1). The trials were mainly conducted across 5 countries: the USA (6 trials), China (3 trials), Israel (1 trial) and one multicenter trial (UK and Sweden). The patients tended to be middle-aged (median age: 59.7 years), with an approximately equal number of men and women (ratio of men to women: 1.1). Eight RCTs used CYP2C9 and VKORC1 genotype testing, two RCTs used CYP2C9 testing and one study used CYP2C9, VKORC1 and CYP4F2 testing. The follow-up periods ranged from 28 days to 3 months. The details of the study characteristics are summarized in Tables 2 and 3.

**Table 2:** Characteristics of RCTs included in the meta-analysis.

Source	Location	Target INR	Genotypes used	Indication	Follow-up	Sample size		Male %		Mean age		Race White%		Dosing method
						PD	CD	PD	CD	PD	CD	PD	CD	
Anderson 2007 [19]	USA	2–3	CYP2C9, VKORC1	PO, DVT, PE, AF, other	3 mo	101	99	49.5	56.6	63.2	58.9	94.1	94.9	PD: regression equation [30]; CD: 10mg warfarin nomogram of Kovacs et al [31]
Borgman 2012 [20]	USA	1.8–3.2	CYP2C9,VKORC1	AF, DVT, stroke, other	12 wk	13	13	54	54	59	45	100	85	PD: PerMIT-guided method [32]; CD: Standard-of-care method [31]
Burmester 2011 [21]	USA	2–3.5	CYP2C9,VKORC1,CYP4F2	arrhythmia, VTE, PV	60 d	115	115	57	61	67.4	69.2	100	100	PD: Marshfield models; CD: guidelines described by Ansell et al [33]
Caraco 2008 [22]	Israel	2–3	CYP2C9	DVT, PE, AF	NA	95	96	48.4	43.8	57.6	59.7	NA	NA	PD: guided by 6 different CYP2C9 genotype-adjusted algorithms; CD: algorithm described by Ageno et al [34]
Hillman 2005 [23]	USA	NA	CYP2C9	AF, PV, PJ, DVT, PE, other	4 wk	18	20	44	45	68.8	70.5	100	100	PD: published multivariate model [35]; CD: 5 mg warfarin/day
Huang 2009 [24]	China	1.8–3	CYP2C9,VKORC1	HV	50 d	61	60	32.8	30	41.6	43	NA	NA	PD: dosing algorithm developed by authors; CD: 2.5 mg/day

**Table 3:** Characteristics of RCTs included in the meta-analysis (Cont).

Source	Location	Target INR	Genotypes used	Indication	Follow-up	Sample size		Male %		Mean age		Race White%		Dosing method
						PD	CD	PD	CD	PD	CD	PD	CD	
Jonas 2013 [25]	USA	2–3 or 2.5–3.5	CYP2C9, VKORC1	AF, DVT, PE, HV, other	90 d	55	54	43.6	50	59	55.3	80	64.8	PD: algorithm developed at the Washington University [36]; CD: same algorithm with clinical data only [36]
Kimmel 2013 [26]	USA	2–3	CYP2C9, VKORC1	DVT, PE, AF, other	28 d	514	501	53	49	59	57	67	66	PD: algorithms included clinical variables and genotype data [36,37]; CD: algorithms included clinical variables only [36,37]
Li 2013 [27]	China	2–3	CYP2C9, VKORC1	PE	50 d	97	95	39.2	40	61.6	60.1	0	0	PD: algorithm developed by authors [27]; CD: initial dose adjusted base on experience
Pirmohamed 2013 [28]	UK and Sweden	2–3	CYP2C9, VKORC1	VTE, AF	3 mo	227	228	63.9	57.9	67.8	66.9	97.8	98.7	PD: a loading-dose algorithm [38]; CD: initial dose based on age, adjusted based on the INR
Wang 2012 [29]	China	1.8–3	CYP2C9, VKORC1	HV	50 d	50	51	30	31.4	41.9	42.8	0	0	PD: With reference to the literature [24]; CD: initiated at 2.5mg/day, and adjusted based on the INR values

PO = Preoperative orthopedic; DVT = deep vein thrombosis; PE = pulmonary embolism; AF = atrial fibrillation; VTE = venous thromboembolism; PV = prosthetic valve; PJ = prosthetic joint; HV: Heart valves; PD = pharmacogenetics-based dosing; CD = conventional dosing; NR = not reported; INR = international normalized

### The risk of bias in the included studies

Randomization was performed in all of the included studies; however, only 6 studies appropriately described the sequence of generation. Most of the studies did not mention allocation concealment, apart from 4 trials. In most of the included RCTs, the participants and personnel were not blinded, corresponding to a high risk of bias. One study showed a high risk of bias in 3 items, and 4 RCTs showed a high risk of bias in 2 items. The risk of bias within the included trials is shown in Table 4.

**Table 4:** Cochrane assessment of bias risk of RCTs.

	Andersonn 2007 [19]	Borgman 2012 [20]	Burmester 2011 [21]	Caraco 2008 [22]	Hillmann 2005 [23]	Huang 2009 [24]	Jonas 2013 [25]	Kimmel 2013 [26]	Li 2013 [27]	Pirmohamed 2013 [28]	Wang 2012 [29]
Random sequence generation	Unclear	Unclear	Low	High	Low	Unclear	Low	Unclear	Low	Low	Low
Allocation concealment	Low	Unclear	Low	Unclear	Low	Unclear	Low	Unclear	Unclear	Unclear	Unclear
Blinding of participants and personnel	Low	High	High	Unclear	High	Unclear	Low	Low	High	High	Unclear
Blinding of outcome assessment	Low	Unclear	Unclear	Unclear	High	Unclear	Low	Low	High	High	Unclear
Incomplete outcome data	Low	High	High	Low	Low	Low	Low	Low	Low	Low	Low
Selective reporting	Low	Unclear	High	Unclear	Unclear	Low	Low	Low	Unclear	Low	Low
Other bias	Low	Unclear	Low	Unclear	Unclear	Low	Low	Low	Unclear	Low	Low

### Primary outcome

#### Time within therapeutic range (TTR)

**Fig 2:**
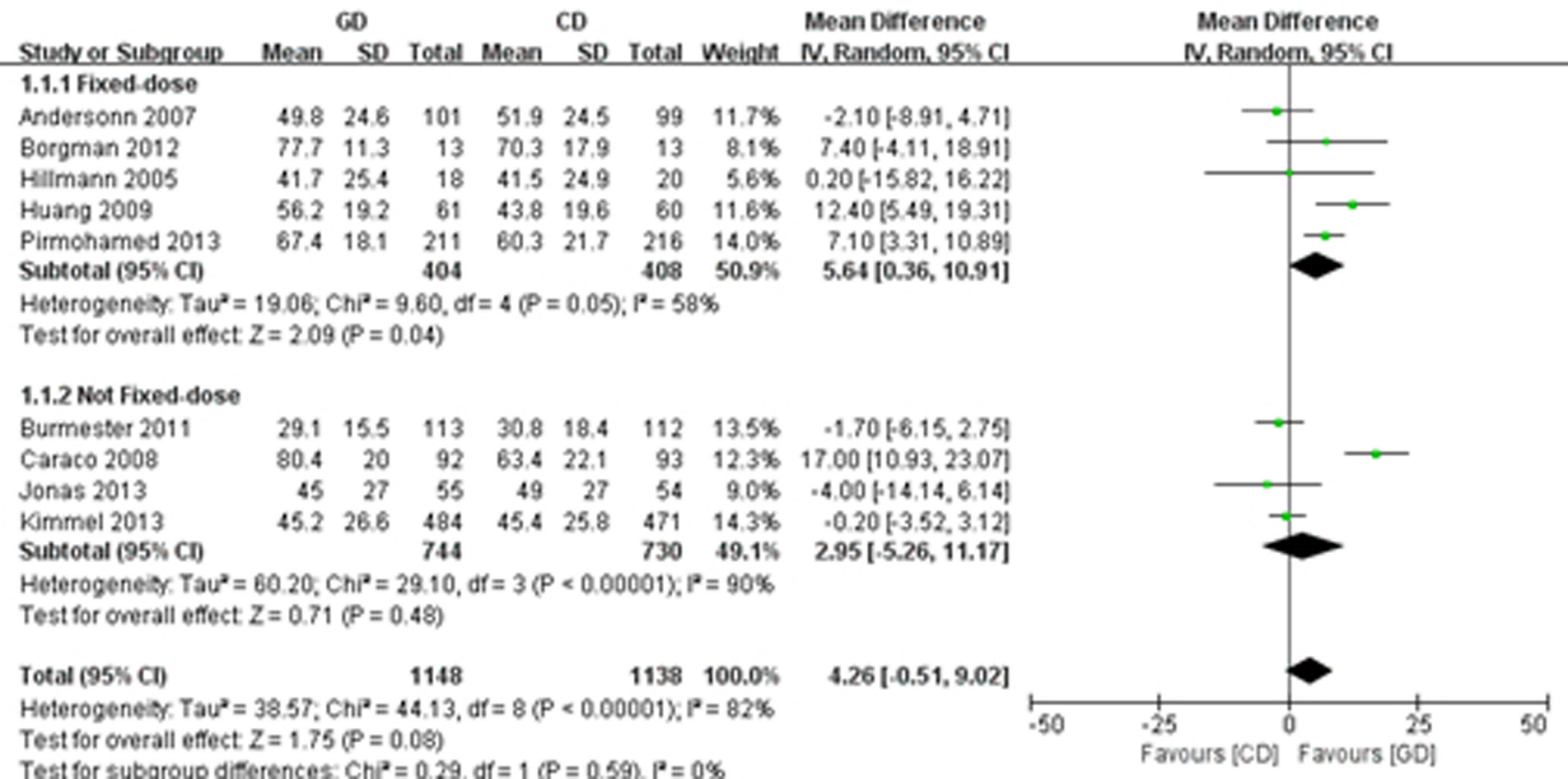
Forest plot of the time within therapeutic range (TTR).

Nine of the trials [19–26,28] reported TTR data. We performed a random effects meta-analysis that included 1,148 participants in the PD group and 1,138 patients in the CD group. The genotype-guided treatment had no significant overall effect on the TTR in comparison to conventional warfarin dosing (MD = 4.26; 95% CI: -5.26 to 11.17; P = 0.08), with significant heterogeneity (I^2^ = 82%, P<0.00001). However, the subgroup analysis showed that the pharmacogenetics-based warfarin dosing group was associated with a higher percentage of TTR than the fixed-dose regimen arm (MD = 5.64; 95% CI: 0.36 to 10.91, P = 0.04), with significant heterogeneity (I^2^ = 58%, P = 0.05), but did not significantly differ from the non-fixed doses regimen arm (MD = 2.95; 95% CI: -5.26 to11.17; P = 0.48), with significant heterogeneity (I^2^ = 90%, P<0.00001) (Fig 2).

### Secondary outcomes

#### INR greater than 4

**Fig 3:**
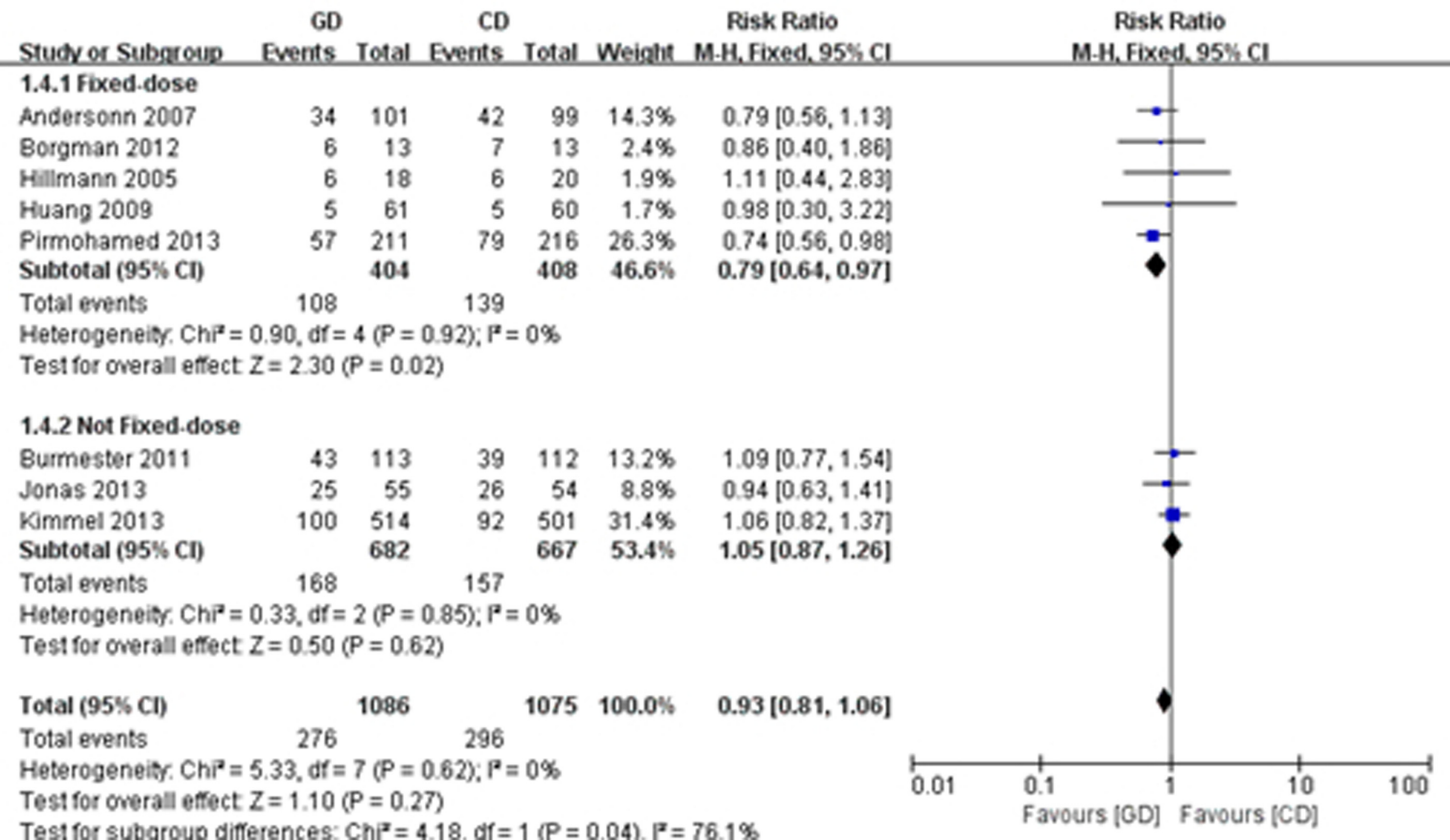
Forest plot of an INR greater than 4.

A total of 8 trials [19–21,23–26,28] reported an INR greater than 4. We carried out a fixed effects meta-analysis that included 1,086 participants in the PD group and 1,075 patients in the CD group. The meta-analysis did not show a significant difference in the frequency of INR greater than 4 between the two arms (RR = 0.93; 95% CI: 0.81 to 1.06; P = 0.27), with a high consistency (I^2^ = 0%, P = 0.62). However, the subgroup analysis showed that the pharmacogenetics-guided group was associated with fewer incidences of INR greater than 4 when compared to the fixed-dose regimen group (RR = 0.79; 95% CI: 0.64 to 0.97; P = 0.02), with a high consistency (I^2^ = 0%, P = 0.92), but did not significantly differ from the non-fixed doses regimen group (RR = 1.05; 95% CI: 0.87 to 1.26; P = 0.62), with no statistical heterogeneity (I^2^ = 0%, P = 0.85) (Fig 3).

#### Time to maintenance dose and time to first therapeutic INR

**Fig 4:**
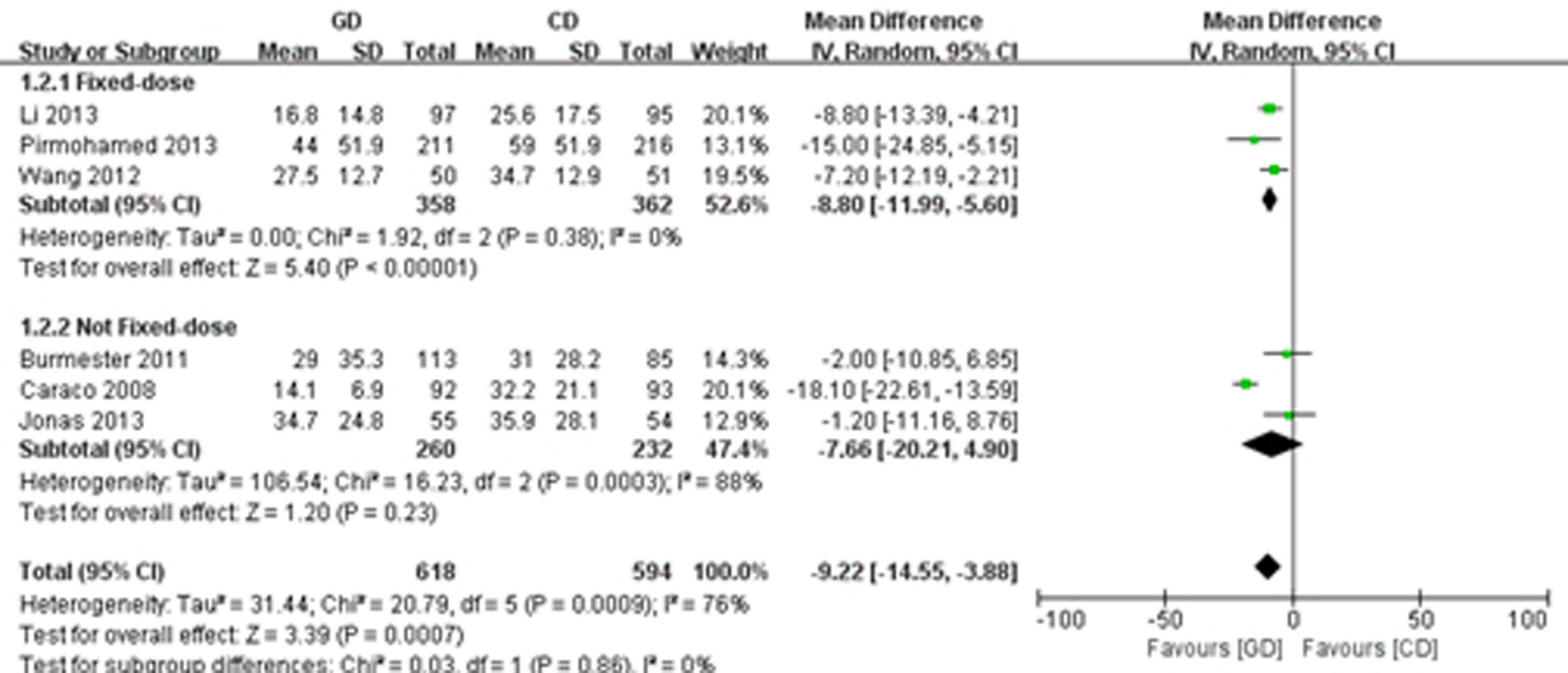
Forest plot of the time to maintenance dose.

**Fig 5:**
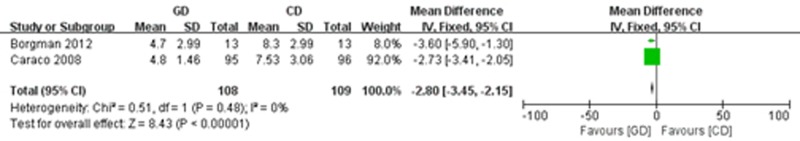
Forest plot of the time to first therapeutic INR.

Six of the trials [21,22,25,27–29] reported on the time to maintenance dose. We carried out a random effects meta-analysis that included 618 participants in the PD group and 594 patients in the CD group. The genotype-guided treatment shortened the time to maintenance dose compared with the conventional regimen group (MD = -9.22; 95% CI: -14.55 to -3.88, P = 0.0007), with significant heterogeneity (I^2^ = 76%, P = 0.0007). The subgroup analysis showed that the time to maintenance dose of the patients in the PD arm was significantly shorter than the fixed warfarin dosing arm (MD = -8.80; 95% CI: -11.99 to -5.60; P<0.00001), with a high consistency (I^2^ = 0%, P = 0.38), but not significantly different from the non-fixed doses regimen group (MD = -7.66; 95% CI: -20.21 to 4.90; P = 0.23]), with significant heterogeneity (I^2^ = 88%, P = 0.0003) (Fig 4). Only two of the studies [20,22] provided the time to first target INR. These results showed that the time to first target INR of patients in the PD group was significantly shorter than that in the CD arm (MD = -2.80; 95% CI: -3.45 to -2.15; P<0.00001), with high consistency (I^2^ = 0%, P = 0.48) (Fig 5).

#### Adverse events

**Fig 6:**
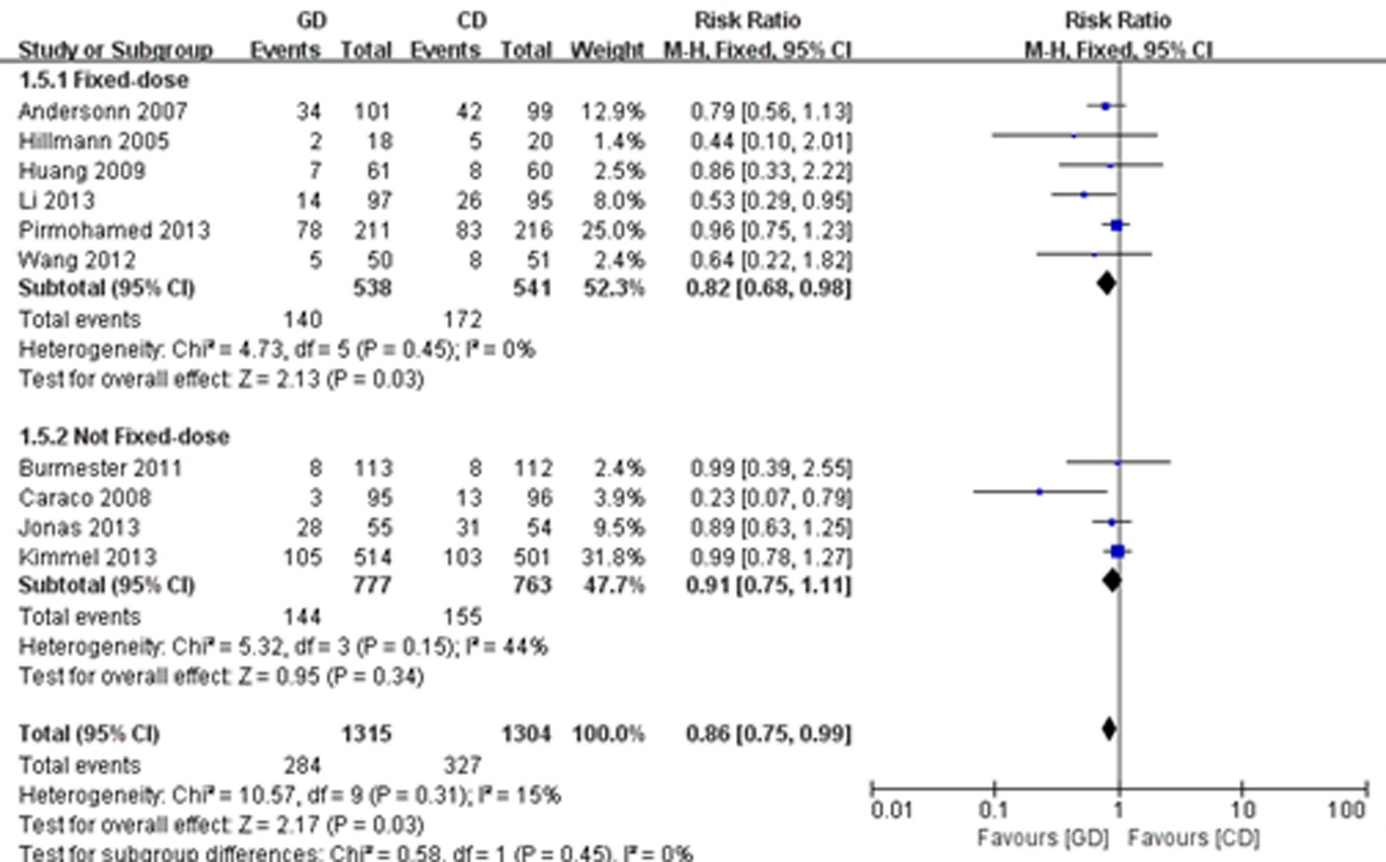
Forest plot of adverse events.

All of the included trials except one [20] reported on adverse events. We carried out a fixed effects meta-analysis that included 1,315 participants in the GD group and 1,304 patients in the CD group. The meta-analysis demonstrated that GD was associated with less adverse events compared with the control group (RR = 0.86; 95% CI: 0.75 to 0.99; P = 0.03), with high consistency (I^2^ = 0%, P = 0.31). The subgroup analysis showed that a reduction in the risk of adverse events was greater in the pharmacogenetics-based group than the fixed warfarin dosing group (RR = 0.82; 95% CI: 0.68 to 0.98; P = 0.03), with high consistency (I^2^ = 0%, P = 0.45); however, there was no difference between the pharmacogenetics-guided dosing group and the non-fixed dosing regimen group (RR = 0.91; 95% CI: 0.75 to 1.11; P = 0.34) (Fig 6).

#### Major bleeding, thromboembolism and all-cause mortality

**Fig 7:**
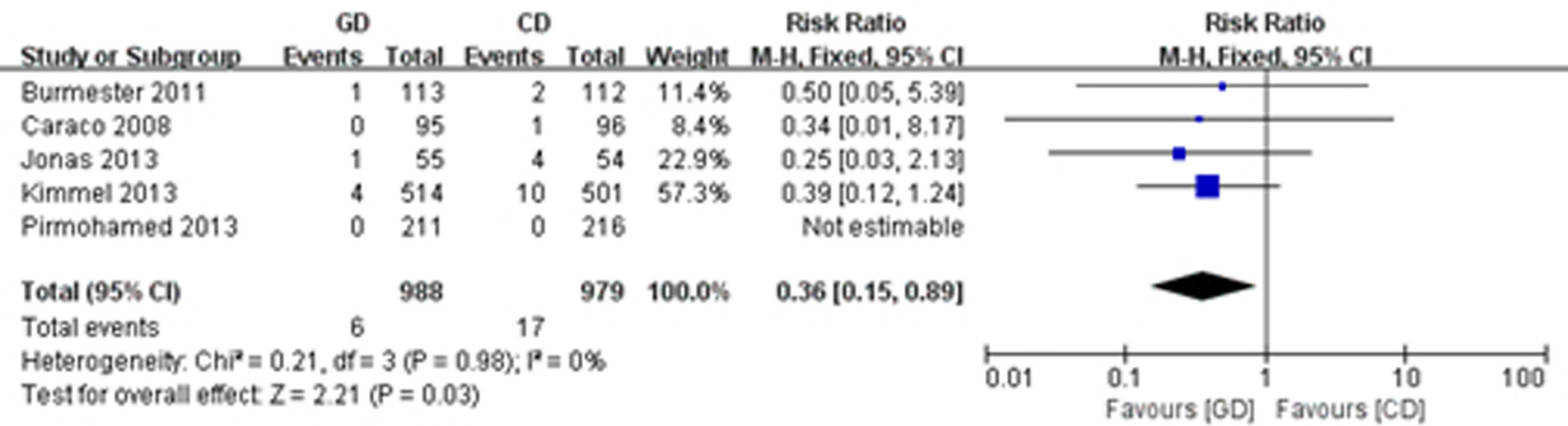
Forest plot of major bleeding.

**Fig 8:**
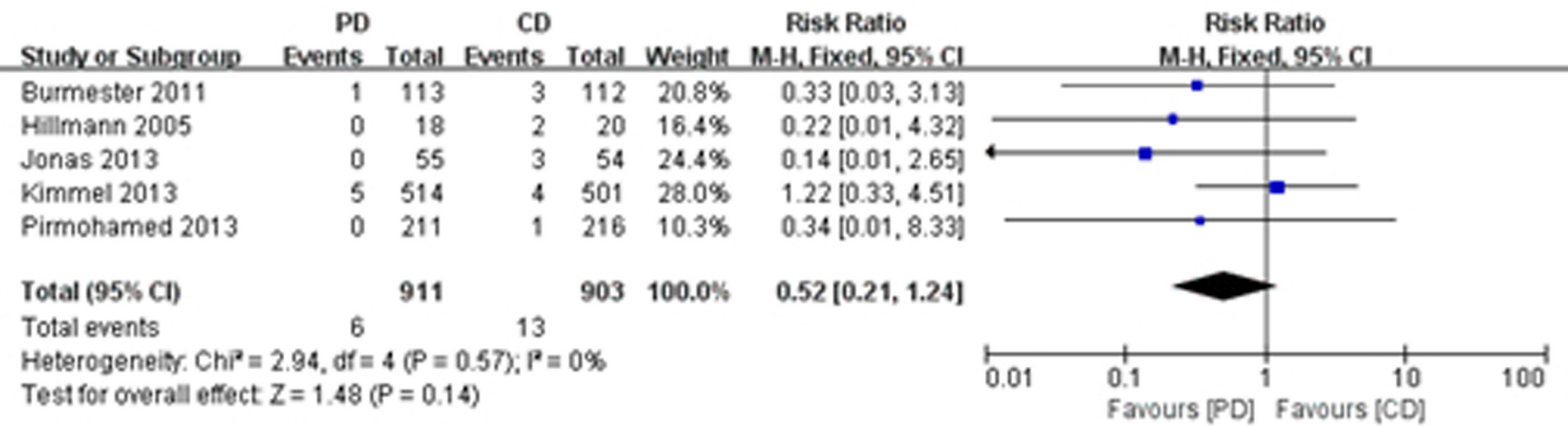
Forest plot of thromboembolism.

**Fig 9:**
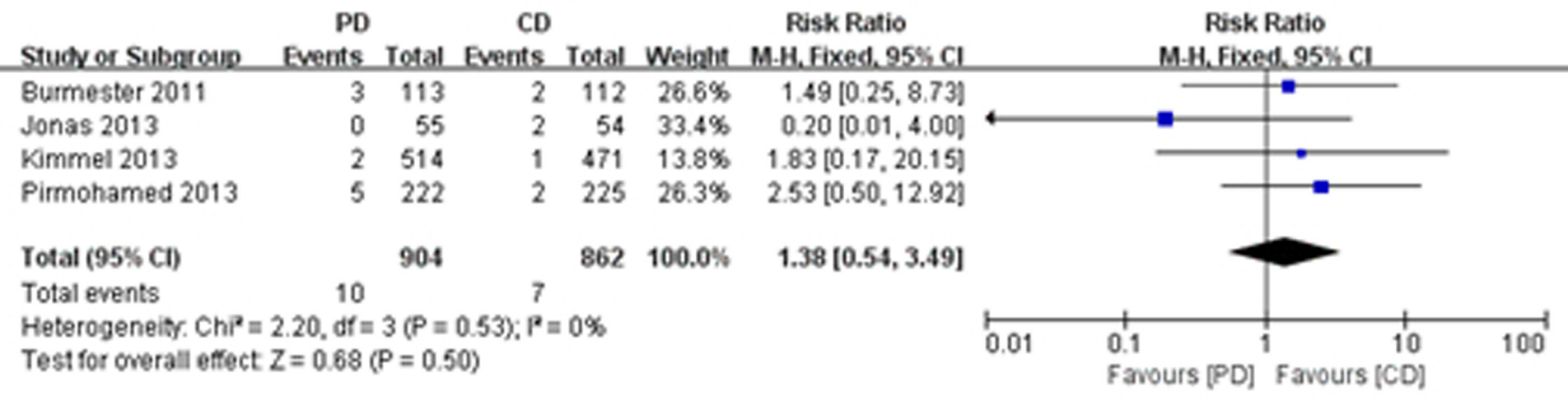
Forest plot of all-cause mortality.

Five of the trials [21,22,25,26,28] reported major bleeding. The meta-analysis showed a significant reduction in the risk of major bleeding (RR = 0.36; 95% CI: 0.15 to 0.89, P = 0.03), with high consistency (I^2^ = 0%, P = 0.98) (Fig 7). A total of 5 studies reported on thromboembolism [21,23,25,26,28], and 4 of the studies [21,25,26,28] reported on all-cause mortality. However, there were no significant differences between treatment groups in terms of the frequency of thromboembolism (RR = 0.52; 95% CI: 0.21 to 1.24, P = 0.14) (Fig 8) and all-cause mortality (RR = 1.38; 95% CI: 0.54 to 3.49, P = 0.50), with no heterogeneity (Fig 9).

### Sensitivity analysis and publication bias

We performed a sensitivity analysis to evaluate the stability of our pooled analysis by excluding the following studies: large trials (the largest trial in our study or subgroup) [26, 28]; small trials (<50 patients) [20, 23]; studies showing a high risk of bias ≥ 2 items [20, 21, 23, 27, 28]; trials including patients with heart valves mainly [24, 29] and the trial using CYP2C9, VKORC1 and CYP4F2 testing [21]. The results indicated that the exclusion of these studies had not changed our primary outcomes (Table 5). Additionally, Egger’s test did not reveal significant evidence of publication bias.

**Table 5:** Sensitivity analysis of time within therapeutic range.

Study omitted	No. of patients	MD (95% Cl)	I^2^
largest trial [26]	955	4.96 (-0.57, 10.49)	81%
large trials [26, 28]	1382	4.48 (-2.55, 11.51)	83%
small trials [20, 23]	64	4.22 (-1.11, 9.56)	86%
high risk of bias ≥ 2 items [20, 21, 23, 27, 28]	716	4.83 (-3.21, 12.86)	89%
including patients with heart valves mainly [24, 29]	121	3.20 (-1.74, 8.13)	81%
using CYP2C9, VKORC1 and CYP4F2 testing [21]	225	5.17 (-0.09, 10.44)	81%

MD = mean difference; CI = confidence interval.

## Discussion

Despite the fact that several models that combine clinical characteristics and genetic information have been shown to accurately predict warfarin doses, these models have not been incorporated into clinical practice because a direct clinical benefit associated with genotype-guided dosing has yet to be demonstrated. As noted in the Food and Drug Administration (FDA) warfarin label, a patient’s CYP2C9 and VKORC1 genotype information can assist in selecting the starting dose; however, the current guidelines of the American College of Chest Physicians (ACCP) recommend against genotype testing for guiding doses of warfarin until more evidence indicates a benefit [39]. Furthermore, several insurance companies and healthcare providers have refused to pay for CYP2C9 and VKORC1 testing for warfarin dosing [40], making the application of genotype testing haphazard and only used in some specialist hospitals.

The present meta-analysis included 11 trials that investigated the efficacy and safety of genotype testing for warfarin dosing guidance. Due to the limited sample size of the published trials, the TTR was frequently used as the primary endpoint [41]. In our study, the TTR was reported in 9 out of 11 trials. Although not statistically significant, we found a trend towards improvement of the percentage of TTR and INR greater than 4 in the genotype-guided group compared with the conventional regimen group.

The time to first target INR and maintenance dose can be used to mirror how quickly effective anticoagulation occurs and once a stable warfarin dose has been established, respectively. In our study, compared with clinical dosing of warfarin, pharmacogenetics-based dosing had significantly shortened the time to maintenance dose and time to first therapeutic INR.

To our knowledge, major bleeding, thromboembolism and adverse events are all clinical endpoints. The definitions of major bleeding and thromboembolism were those used in individual trials. We observed that the pharmacogenetics-based dosing of warfarin could be beneficial for the improvement of clinical endpoints, with a risk reduction for major bleeding by 64% and adverse events by 14%. Although not statistically significant, we found a trend towards a reduction in thromboembolism events in the genotype-guided group. A shortened TTR was likely to be the main mechanism responsible for the improvement of these clinical outcomes, and the lack of statistical significance for all-cause mortality may be due to the limited sample size.

Recently, several meta-analyses have reported the effects of pharmacogenetics- based warfarin dosing on clinical outcomes [42–46]. These meta-analyses have included different numbers of trials and participants, and thus the conclusions have been inconsistent. The first published meta-analysis of pharmacogenetics-based versus clinical dosing of warfarin with 3 RCTs observed no statistically significant difference in the hemorrhage rate and TTR [42]. A previously published meta-analysis including 10 RCTs reported that genotype-based dosing presented some benefits. In particular, the authors found that genotype-based warfarin dosing increased the rate of TTR and reduced the risk for bleeding complications [43]; however, a non-randomized trial described by Lenzini PA et al. [47] was inappropriately included in this meta-analysis. A meta-analysis conducted by Franchini M et al. [44] including 2,812 patients indicated that pharmacogenetics-based initial vitamin K antagonist dosing is able to reduce major bleeding by approximately 50% compared with clinically based methods; however, their study included oral anticoagulants not limited to warfarin. Recently, a meta-analysis [45] including 10 RCTs showed that compared to warfarin therapy with a short time duration (within 1 month), long-time (>1 month) anticoagulation suggested a superior effect; however, a cohort study described by McMillin GA [48] was inappropriately included in this study. A meta-analysis conducted by Stergiopoulos K et al. [46] including 9 RCTs reported that a genotype-based dosing strategy did not result in a greater percentage of TTR, fewer patients with an INR greater than 4, or a reduction in major bleeding or thromboembolism events compared with conventional dosing of warfarin. However, this meta-analysis did not include any trials involving Chinese or East Asian patients as the main research subjects. Our study includes more Chinese patients than previous studies as shown above. These trials can help to evaluate the feasibility of genetics-based warfarin dosing in Chinese population.

We defines I^2^ >50% to represent significant heterogeneity. There are two endpoints (TTR and the percentage of INR greater than 4) represented significant heterogeneity. Other outcomes represented low heterogeneity (I^2^ = 0). In the subgroup analysis of the present study, genotype-based dosing was associated with a significant improvement in the rate of TTR compared with the fixed initial dose group but failed to show a significant difference compared to the non-fixed dosage group. The percentage of INR greater than 4 presented a similar result. We found that the pharmacogenetics-based arm was associated with an approximately 21% lower percentage of INR greater than 4 compared with the fixed-dose regimen. Therefore, we suggest that genotype-guided dosing should be applied rather than a fixed-dose regimen.

CYP4F2 was first identified as a contributor to warfarin dosing in Europeans, but without large effects or much compelling data [49]. Only one trial using CYP4F2 testing described by Burmester JK et al. [21] was included in our meta-analysis. We performed a sensitivity analysis to evaluate the stability of our meta-analysis by excluding that trial, and no inconsistency results were revealed.

Although several new oral anticoagulants (dabigatran, apixaban, rivaroxaban, and most recently edoxaban) have been developed, warfarin will continue to be used and will likely remain a mainstay therapy for the following reasons: first, the long-term safety of these new oral anticoagulants remains unclear. Second, their application is limited or contraindicated under several circumstances. For instance, none of these new oral anticoagulants is approved for use during pregnancy or in babies and children. Additionally, new oral anticoagulants have not yet been applied in patients with mechanical prosthetic heart valves. Furthermore, no head-to-head trial between genotype-guided dosing of warfarin and new oral new oral anticoagulants has been performed, although this type of study should be conducted in the future.

## Limitations

There are several limitations to our study. First, to improve the quality of the data collected, only 11 RCTs were included in our study, although we identified a significant number of non-RCTs. Our study may also not be representative of patients seen in daily practice. Second, the RCTs in our review varied by population, length of follow-up, genotype-based and single clinical algorithm. The definitions of outcomes were also inconsistent across studies. Third, the largest trial in our study [26] contributed to almost 40% of the sample. In addition, the largest trial in the fixed-dose group reported by Pirmohamed et al. [28] contributed to almost 50% of the sample. These large trials may have induced bias in evaluating the endpoints in our study.

## Supporting Information

S1 PRISMA Checklist PRISMA checklist. (DOC)

S1 Appendix Search strategy. (DOCX)
