# Supplementary material for: Pharmacogenetics-Based versus Conventional Dosing of Warfarin: A Meta-Analysis of Randomized Controlled Trials
Source: PLoS One. 2015 Dec 16;10(12):e0144511. doi: 10.1371/journal.pone.0144511 (PMC4682655; doi:10.1371/journal.pone.0144511)
Supplement: S1 Appendix — (DOCX) [file pone.0144511.s002.docx]

Table S1. Search strategy used in Embase database.

| **Search number** | **Search query** |
| --- | --- |
| #1 | ‘randomized controlled trial’/exp |
| #2 | ‘randomized controlled trial’ |
| #3 | random* |
| #4 | #1 OR #2 OR #3 |
| #5 | ‘genotype’/exp |
| #6 | genotype |
| #7 | ‘genes’/exp |
| #8 | genes |
| #9 | ‘alleles’ /exp |
| #10 | alleles |
| #11 | genetic |
| #12 | ‘pharmacogenetics’/exp |
| #13 | pharmacogenetics |
| #14 | ‘genomics’ /exp |
| #15 | genomics |
| #16 | #5 OR #6 OR #7 OR #8 OR #9 OR #10 OR #11 OR #12 OR #13 OR #14 OR #15 |
| #17 | warfarin |
| #18 | ‘vitamin k antagonists’ |
| #19 | ‘vitamin k antagonist agent’ |
| #20 | #17 OR #18 OR #19 |
| #21 | #4 AND #16 AND #20 |

Table S2. Search strategy used in Cochrane Library.

| **Search number** | **Search query** |
| --- | --- |
| #1 | genotype |
| #2 | genes |
| #3 | alleles |
| #4 | polymorphism |
| #5 | pharmacogenetics |
| #6 | genomics |
| #7 | pharmacogenomics |
| #8 | #1 OR #2 OR #3 OR #4 OR #5 OR #6 OR #7 |
| #9 | warfarin |
| #10 | vitamin k antagonists |
| #11 | vitamin k antagonist agent |
| #12 | #9 OR #10 OR #11 |
| #13 | #8 AND #12 |

Table S3. Search strategy used in China National Knowledge Infrastructure (CNKI).

| **Search number** | **Search query** |
| --- | --- |
| #1 | 基因型 |
| #2 | 基因多态性 |
| #3 | 遗传药理学 |
| #4 | 单核苷酸多态性 |
| #5 | 药物基因组学 |
| #6 | #1 OR #2 OR #3 OR #4 OR #5 |
| #7 | 华法林 |
| #8 | 华法令 |
| #9 | #7 OR #8 |
| #10 | 随机对照试验 |
| #11 | RCT |
| #12 | #10 OR #11 |
| #13 | #6 AND #9 AND #11 |

Table S4. Search strategy used in VIP database.

| **Search number** | **Search query** |
| --- | --- |
| #1 | 基因型 |
| #2 | 基因多态性 |
| #3 | 药物遗传学 |
| #4 | #1 OR #2 OR #3 |
| #5 | 华法林 |
| #6 | 华法令 |
| #7 | #5 OR #6 |
| #8 | #4 AND #7 |

Table S5. Search strategy used in Wan-fang database.

| **Search number** | **Search query** |
| --- | --- |
| #1 | 基因型 |
| #2 | 基因多态性 |
| #3 | 药物遗传学 |
| #4 | #1 OR #2 OR #3 |
| #5 | 华法林 |
| #6 | 华法令 |
| #7 | #5 OR #6 |
| #8 | #4 AND #7 |
